# Insulin resistance, selfish brain, and selfish immune system: an evolutionarily positively selected program used in chronic inflammatory diseases

**DOI:** 10.1186/ar4688

**Published:** 2014-11-13

**Authors:** Rainer H Straub

**Affiliations:** 1 Laboratory of Experimental Rheumatology and Neuroendocrine Immunology, Division of Rheumatology, Department of Internal Medicine, University Hospital, 93042 Regensburg, Germany

## Abstract

Insulin resistance (IR) is a general phenomenon of many physiological states, disease states, and diseases. IR has been described in diabetes mellitus, obesity, infection, sepsis, trauma, painful states such as postoperative pain and migraine, schizophrenia, major depression, chronic mental stress, and others. In arthritis, abnormalities of glucose homeostasis were described in 1920; and in 1950 combined glucose and insulin tests unmistakably demonstrated IR. The phenomenon is now described in rheumatoid arthritis, systemic lupus erythematosus, ankylosing spondylitis, polymyalgia rheumatica, and others. In chronic inflammatory diseases, cytokine-neutralizing strategies normalize insulin sensitivity. This paper delineates that IR is either based on inflammatory factors (activation of the immune/ repair system) or on the brain (mental activation via stress axes). Due to the selfishness of the immune system and the selfishness of the brain, both can induce IR independent of each other. Consequently, the immune system can block the brain (for example, by sickness behavior) and the brain can block the immune system (for example, stress-induced immune system alterations). Based on considerations of evolutionary medicine, it is discussed that obesity *per se *is not a disease. Obesity-related IR depends on provoking factors from either the immune system or the brain. Chronic inflammation and/or stress axis activation are thus needed for obesity-related IR. Due to redundant pathways in stimulating IR, a simple one factor-neutralizing strategy might help in chronic inflammatory diseases (inflammation is the key), but not in obesity-related IR. The new considerations towards IR are interrelated to the published theories of IR (thrifty genotype, thrifty phenotype, and others).

## Introduction

In 1916, the diabetologist Elliott P Joslin recognized that 'hyperglycemic situations appear after infectious diseases, painful conditions such as gall stones, and trauma' [1]. In 1920, Pemberton and Foster described impaired glucose regulation in soldiers with arthritis [2]. In 1924, Rabinowitch observed that diabetic patients need much more insulin during infection [3]. In 1929, Root summarized the presence of an inadequately high need for insulin in different diseases, and he called the phenomenon 'insulin resistance' (IR) [4].

Over the last century, IR was found in physiological states, disease states, and diseases such as diabetes mellitus, obesity, infection, sepsis, arthritis of different types (including rheumatoid arthritis (RA)), systemic lupus erythematosus, ankylosing spondylitis, trauma, painful states such as postoperative pain and migraine, schizophrenia, major depression, and mental stress, to name the most important (chronology of events is summarized in Table 1). IR thus seems to be present in many diseases states outside the field of diabetology or - more specifically - exterior of inherited IR syndromes (called the type A syndrome of IR) and also beyond autoantibodies to insulin or insulin receptor (type B syndrome of IR) [5].

**Table 1:** History of insulin resistance from different perspectives of research in the fields of diabetology, infection/inflammation, pain, mental activation, trauma, and rheumatology.

Year	Author	Phenomena	Reference
1916	Joslin	Hyperglycemia in infectious diseases,^a ^painful gallstones,^b ^trauma^c^	[1]
1920	Pemberton and Foster	Impaired glucose regulation in soldiers with arthritis^a^	[2]
1924	Rabinowitch	Enormous doses of insulin needed in infected diabetic patients^a^	[3]
1929	Root	IR in the context of different diseases^a,b,c^	[4]
1936	Himsworth and Kerr	Insulin-sensitive and insulin-insensitive diabetes	[106]
1938	Thomsen	Traumatic diabetes^c^	[107]
1938	Warren	β-cell defects in older longstanding diabetic patients	In [108]
1950	Liefmann	IR in rheumatoid arthritis (combined glucose and insulin test)^a^	[16]
1956	Arendt and Pattee	IR in obese subjects	[109]
1957	Collins	IR in schizophrenia^d^	[110]
1960	Yalow and Berson	IR in diabetic subjects (high glucose despite high insulin)	[24]
1963	Randle and colleagues	Fatty acids support IR	[25]
1965	van Praag and Leijnse	Major depression induces IR^d^	[111]
1965	Butterfield and Wichelow	Forearm insulin sensitivity test	[112]
1970	Shen and colleagues	Quadruple insulin sensitivity test	[113]
1979	DeFronzo and colleagues	Euglycemic insulin clamp technique in combination with radioisotope turnover, limb catheterization, indirect calorimetry, and muscle biopsy	[114]
1979	Wolfe	Review: sepsis and trauma induce IR^a,b,c^	[115]
1982	Kasuga and colleagues	Insulin induces tyrosine phosphorylation of the insulin receptor	[116]
1982	Ciraldi and colleagues	Reduced insulin-stimulated glucose uptake in type 2 diabetes	[117]
1984	Grunberger and colleagues	Dissociation between normal insulin binding and defective tyrosine kinase activity of the insulin receptor	[118]
1986	Garvey and colleagues	Hyperinsulinemia induces insulin receptor desensitization	[119]
1987	Svenson and colleagues	IR in rheumatoid arthritis^a^	[17]
1988	Krieger and Landsberg	Hypertension, hyperinsulinemia, insulin resistance and SNS	[120]
1988	DeFronzo	Hyperglycemia decreases glucose transport and inhibits beta-cell function (glucotoxicity)	[121]
1988	DeFronzo, Reaven	Increased free fatty acids play key role in IR, β-cell dysfunction, and hepatic gluconeogenesis (lipotoxicity)	[121,122]
1988	Uchita and colleagues, Greisen and colleagues	Pain influences IR via the HPA axis and SNS^b^	[123,124]
1992	Feingold and Grunfeld	Cytokines like TNF play a role in hyperlipidemia and diabetes^a^	[125]
1993	Hotamisligil and colleagues	TNF critically influences IR^a^	[34]
1994	Moberg and colleagues	Mental stress induces acute IR in type 1 diabetic patients^d^	[126]
1996	Keltikangas and colleagues	Mental stress is accompanied by IR in nondiabetic people^d^	[127]
1999	Björntrop	IR as a consequence of exaggerated HPA axis and SNS activation (CNS stress is the trigger)^d^	[28]
2000	Chrousos	Mental stress-induced hypercortisolism induces IR (the pseudo-Cushing state)^d^	[29]
2000	Seematter and colleagues	Mental stress acutely increases insulin-stimulated glucose utilization in healthy lean humans but not in obese nondiabetic humans^d^	[128]
2004	Tso and colleagues	Patients with systemic lupus erythematosus demonstrate IR independent of autoantibodies to insulin receptor^a^	[19]
2005	Kiortsis and colleagues, Stagakis and colleagues	Patients with ankylosing spondylitis and rheumatoid arthritis have IR, which is reduced after anti-TNF therapy^a^	[20,44]
2007	Larsen and colleagues	IL-1ra improved beta-cell secretory function in type 2 diabetic patients (no influence on IR)^e^	[129]
2008	Fleischman and colleagues, Goldfine and colleagues	Salsalate improved insulin sensitivity in young obese adults and in type 2 diabetic patients	[43,130]
2010	Schultz and colleagues	Patients with rheumatoid arthritis show IR, which can be reduced by blocking IL-6^a^	[45]
2012, 2014	DIAGRAM and colleagues, Fall and Ingelsson	Human gene polymorphisms link both inflammation and metabolic disease	[93,131]

CNS, central nervous system; DIAGRAM, DIAbetes Genetics Replication And Meta-analysis Consortium; HPA, hypothalamic-pituitary-adrenal; IL, interleukin; IR, insulin resistance; OGTT, oral glucose tolerance test; SNS, sympathetic nervous system; TNF, tumor necrosis factor. ^a^Insulin resistance as a consequence of infection or inflammation. ^b^Insulin resistance as a consequence of pain. ^c^Insulin resistance as a consequence of trauma. ^d^Insulin resistance as a consequence of mental activation. ^e^Approved by the US Food and Drug Administration for patients with type 2 diabetes mellitus.

When considering these diseases and disease states, one observes two major clusters of clinical entities that are linked to IR: inflammation with an activated immune/ repair system; and increased mental activation. In this clearly defining distinction, obesity and type 2 diabetes mellitus (T2D) can be integrated into the first cluster due the inflammatory aspect of IR in these entities [6-9]. However, obesity and consequently T2D might also be integrated into the latter cluster because chronic mental stress is a well-known forerunner of obesity in approximately 40% of investigated stressed subjects [10-15]. At this point the question is why these two disease clusters are linked to IR, which will be addressed in the present paper.

Since chronic inflammatory diseases (CIDs) such as arthritis were among the first to be linked to IR [2,16], newer work in rheumatology has recognized IR in many CIDs [17-20], cytokine-neutralizing strategies decrease IR in CIDs [20-22], and CID patients are at increased risk to develop T2D [23], the special view from rheumatology to IR is understandable and necessary. The reader will see that IR is not an endocrine disorder *per se*, but more a disorder of several systems, better tackled from an inter-disciplinary standpoint of neuroendocrine immunology.

## Features of insulin resistance and pathophysiology

Originally, IR was defined as a subnormal biologic response to a certain insulin concentration, whereby the word subnormal already suggests illness. In the late 1950s, Yalow and Berson developed the radioimmuno-assay to measure circulating insulin in the blood. In this early paper, they described a state of IR in T2D patients: '... [there is a] lack of responsiveness of blood sugar, in the face of apparently adequate amounts of insulin secreted ...' [24]. The classical characteristics of IR are presented in Table 2. Elements given in this table work together to induce clinically observed hyperglycemia and very low density lipoprotein hyperlipidemia (triglycerides) despite elevated insulin levels.

**Table 2:** Classical signs of insulin resistance until 1995 [5,28,121,122].

Structure, organ	Observed change
Insulin receptor	Inhibited
Insulin receptor signaling cascade	Inhibited
Muscle	
Glycogen synthase	Inhibited
Hexokinase II	Inhibited
Pyruvate dehydrogenase	Inhibited
Liver	
Hepatic glucose production (gluconeogenesis, glycogenolysis)	Stimulated
Insulin clearance	Stimulated
Adipose tissue	
Free fatty acid mobilization	Stimulated
Signs in circulating blood	
Hyperglycemia	Yes
Hyperlipidemia^a^	Yes
Glucagon	Increased

^a^Triglyceride-free fatty acid-very low density lipoprotein-triglyceride cycle.

IR is measured by different techniques, whereby the gold standard is the hyperinsulinemic euglycemic clamp and the silver standard is the frequently sampled intravenous glucose tolerance test (Table 3). To study IR or insulin sensitivity in CIDs, simple fasting indices are often used such as the homeostasis model assessment insulin resistance and the Quicki (Table 3), which are adequate when applied in larger clinical studies.

**Table 3:** Methods to measure insulin resistance.

Technique	Notes	Reference
Reference methods		
Hyperinsulinemic euglycemic clamp	Gold standard, highly invasive	[114]
Frequently sampled intravenous glucose tolerance test	Silver standard, invasive	[132]
Oral glucose tolerance test		
Insulin sensitivity glycemic index = 1 + 2/INSp × GLYp)	Most commonly used, little invasive	[133]
Whole body insulin sensitivity	Little invasive	[133]
Muscle IS = (Δglucose/Δtime)/mean plasma insulin^a^	Little invasive	[134]
Hepatic IS = glucose_0-30 minutes_[AUC] × insulin_0-30 minutes_[AUC]^b^	Little invasive	[134]
Fasting simple indices		
Homeostasis model assessment insulin resistance (HOMA-IR)	Non-invasive	[133]
Newer version of the HOMA-IR (HOMA2-S)	Non-invasive	[133]
FGIR = fasting glucose (mg/dl)/fasting insulin (mU/l)	Non-invasive	[133]
Quicki = 1/(log fasting insulin (mU/l) + log fasting glucose (mg/dl))	Non-invasive	[133]
Biochemical markers of insulin resistance		
Sex hormone binding globulin	Non-invasive	[133]
Insulin-like growth factor binding protein 1	Non-invasive	[133]
Other markers: YKL-40, alpha-hydroxybutyrate, soluble CD36, leptin, resistin, interleukin-18, retinol binding protein-4, and chemerin	Non-invasive	[135]

AUC, area under the curve; GLYp, area under glucose curve; INSp, area under the insulin curve; IS, insulin sensitivity. ^a^The rate of decay of plasma glucose concentration from its peak value to its nadir (Δglucose/Δtime) during the oral glucose tolerance test. ^b^The product of the total AUC for glucose and insulin during the first 30 minutes of the oral glucose tolerance test.

### Pathophysiology of insulin resistance - a chronology of models

The first viable theory on IR was presented by Randle, who suggested that IR in muscle and adipose tissue is based on the glucose-fatty acid cycle [25]. The theory suggested that IR is a consequence of an increased presence of circulating fatty acids and ketone bodies that lead to defects in glucose utilization and an ever- increasing insensitivity to insulin. The biochemical principles of this model are still valid and useful today.

Further clarification throughout the 1960s and 1970s came from endocrine diseases that were accompanied by IR. The explanatory power of hormones is particularly obvious in diseases with an overproduction of a distinct glucogenic hormone such as in Cushing's syndrome (cortisol), acromegaly (growth hormone), pheochromocytoma (catecholamines), glucagonoma, thyrotocicosis (thyroxine, triiodothyronine), and insulinoma (IR as a consequence of insulin receptor desensitization) [5]. Since these diseases were accompanied by IR, the respective hormones became the focus of IR research (called the insulin antagonists; not to speak of antibodies to insulin or insulin receptor). However, in the diseases mentioned in Table 1, IR was not accompanied by enormous serum levels of hormones as in these endocrine tumors.

Physiological conditions and disease states with upregulated stress hormones were found to be accompanied by IR, such as in psychological stress, psychiatric disease, starvation, fasting, and others (Table 1). The activation of stress axes is very closely related to the abovementioned cluster of mental activation. For example, an overactive stress system has been described in different forms of IR [26,27]. Stress system activation is an explanatory model for IR, still *in vogue *[28-33], but in 1993 the mainstream of research turned to inflammation-related IR (discussed in the paragraphs following the next paragraph) [34].

In addition, several authors indicated the central role of the brain because it dictates nutrient intake and foraging behavior. Excess energy intake *per se *would be an important factor for obesity and, thus, a possible cause of subsequently developing IR. This has been demonstrated in humans to play a role in congenital severe obesity with congenital leptin deficiency [35], or a mutation in the melanocortin receptor type 4 [36]. There is a highly delicate system of hypothalamic regulation of satiety versus food intake, which is influenced by distinct pathways within the brain and from the periphery [31,37]. Close relationships exist with psychological components comprising mood disturbances, altered reward perception and motivation, or addictive behavior [38]. The interested reader is referred to comprehensive reviews of the subject [31,38,39].

Nowadays, inflammation-mediated IR is another important explanatory platform of IR in adipocytes, myocytes, and hepatocytes [7,34,40,41]. Disruption of insulin signaling at the level of insulin receptor substrate-1 and insulin receptor substrate-2 and further downstream by tumor necrosis factor (TNF) signaling, toll-like receptor signaling, nuclear factor-κB and inhibitor of nuclear factor-κB, and FoxO1 activation are key elements of inflammation-related IR [6,40,42]. Crucial cytokines in IR are TNF, interleukin (IL)-1β, IL-6, IL-18, and adipokines. Although the concept behind inflammation-related IR is convincing, neutralization of TNF or IL-1β had no influence on IR in obese patients or T2D patients [40]. This might depend on the redundancy of cytokine pathways because, typically, only one cytokine is neutralized while many cytokines act in parallel. This might be overcome by a broader inhibition of proinflammatory signaling pathways, which has been shown for salsalate therapy that reduced IR in patients with T2D [43]. In patients with CIDs, TNF and IL-6 neutralizing strategies reduced IR [20,44,45]. Until now it is not clear why the neutralizing strategies perfectly improve insulin sensitivity in CIDs but not in patients without CIDs. This discrepancy will be discussed in a model of IR that integrates the findings of CID patients (see below).

In addition to the cytokine-centered theory of IR, a relatively new aspect is nutrient-induced inflammation that leads to endoplasmic reticulum stress, activation of jun-N-terminal kinase, and inhibition of insulin receptor substrate-1 and AKT (v-akt murine thymoma viral oncogene homolog 1) and, thus, IR in the liver and adipose tissue [6]. In this model of metaflammation (metabolic inflammation), free fatty acids can activate toll-like receptors, and free fatty acids and glucose undergoing oxidation in mitochondria stimulate free radical production, both of which inhibit insulin signaling [6,46]. The theory describes that nutrient overload in our modern society of affluence gradually increases the involvement of immune system pathways. This leads to ongoing inflammation, mainly in fat tissue as substantiated by leukocyte infiltration (the macrophage is the big player). In consequence, involvement of these inflammatory pathways intensifies the inhibition of metabolic pathways [6]. In addition, in patients with obesity, changes of the gut microbiota were observed, which in itself can be an inflammatory factor that contributes to IR [47-49].

In this short pathophysiology collection of IR, we recognize again the two clusters linked to IR: inflammation with an activated immune/repair system; and increased mental activation (mood, food intake, stress and stress axes). However, the appearance of the two clusters is not yet explained by the interplay of the abovementioned pathophysiological elements. Possibly, published theories on IR with an evolutionary perspective might help to explain the two clusters.

### Evolutionary medicine - theories of insulin resistance, 1962 to 2014

The theories of IR are summarized in Table 4 and are shortly recapitulated here. The thrifty genotype hypothesis of 1962 states that a gene has been positively selected for an exceptionally efficient intake and utilization of food, which was good for hunter-gatherers in a feast/ famine environment but is not good for modern people in a world of plenty. In the original theory, a single gene was made responsible for rapid postprandial insulin release that supported quick storage of energy-rich substrates (called the quick insulin trigger) [50,51]. While the original theory focused on the quick insulin trigger, an alternative model focused on possible genes involved in IR [52]. Today, we know that obesity and IR are based on a polygenic background with many single nucleotide polymorphisms with small effect sizes. Selection on such mutations would probably be very weak because the individual advantages they would confer would be very small. The theory has been criticized due to modest support by genetic analyses; it has been even rejected, but it is still in use and has been adapted by researchers in the field of eating disorders [53].

**Table 4:** Characteristics of theories on insulin resistance as observed from an evolutionary medicine standpoint.

Theory of insulin resistance	Year	Reference
Thrifty genotype hypothesis: quick hyperinsulinemia after food intake to store energy in fat tissue and elsewhere (quick insulin trigger)	1962, 1999	[50,51]
(Not so) Thrifty genotype hypothesis: starvation induces a special form of IR in order to conserve nitrogen (= amino acids from muscle and elsewhere)a	1979	[54]
Thrifty phenotype hypothesis: intrauterine constraints induces IR and insulin deficiency, which allows the organism to survive long enough to reproduce in a nutritionally deprived environment but which leads to obesity in a world of plenty; maternal constraints support IR (small mother, first baby, many babies in parallel, maternal undernutrition, and similar)	1992, 2001	[55,56]
Based on the thrifty genotype hypothesis: an insulin resistance genotype and a cytokine genotype exist (much IR, high cytokine response); IR is helpful for infections	1999	[61]
Refined thrifty phenotype theory: predictive adaptive response model: the relative difference in nutrition between the prenatal and postnatal environment, rather than an absolute level of nutrition, determines the risk of IR	2004	[60]
Central resistance model: there exists a homeostatic regulation of weight gain versus weight loss but defects in the weight loss system leads to obesity (for example, insulin and leptin signaling, SOCS3, PTB-1B)	2004	[64]
Thrifty genotype plus breakdown of robustness: the basis is the thrifty genotype model; a robust glucose control system evolved during evolution, the breakdown of which induces positive disease-stabilizing feedback loops (TNF)	2004	[63]
Thrifty genotype: integration of cellular pathogen-sensing and nutrient-sensing pathways (cytokines, TLRs, JNK, Ikkβ, PKC, ER stress)	2006	[62]
Good calories-bad calories hypothesis: wrong nutrients, particularly carbohydrates, lead to obesity and IR; a paleolithic diet has quite different qualities that prevents obesity and western diseases	2010, 2012	[65,66]

ER, endoplasmic reticulum; Ikkβ, inhibitor of nuclear factor-κB kinase β; IR, insulin resistance; JNK, jun-N-terminal kinase; PKC, protein kinase C; PTB-1B, protein tyrosine phosphatase 1B; SOCS3, suppressor of cytokine signaling 3; TLR, toll-like receptor; TNF, tumor necrosis factor. aThis is a special form of IR without hyperinsulinemia on the basis of a strong response of counterregulatory hormones. It is questionable to call it IR because of missing hyperinsulinemia and missing inflammation. In addition, activity of the sympathetic nervous system is low while activity of the hypothalamic-pituitary-adrenal axis is high in the typical nadir.

Another theory of starvation-induced IR proposes that IR of the muscle during fasting is a positively selected program to maintain high circulating glucose levels in order to protect muscle from proteolysis during starvation [52,54]. In addition, during starvation, lipolysis is switched on, leading to provision of free fatty acids and then ketone bodies that can be used by the brain. Both mechanisms spare glucose and glucogenic amino acids in the muscle. IR in the context of starvation is of a special form because insulin levels are very low, no inflammation accompanies starvation, and counterregulatory hormones such as glucagon and cortisol are continuously upregulated. This situation does not apply to IR observed in CIDs and obesity because hyperinsulinemia and inflammation are a hallmark.

Another important theory of IR is the thrifty phenotype hypothesis [55,56]. This model is based on the important observations that underweight babies more often develop IR and obesity compared with normal weight children. In this theory, intrauterine malnutrition and other fetal constraints induce insulin deficiency (lack of the growth-promoting activities of insulin) and a postnatal state of regulatory IR, which leads to rapid postnatal increase of adipose tissue that remains stable throughout life (accompanied by cardiovascular disease in the older person, and so forth) [57]. In many studies all over the world, the epidemiological findings were very supportive of the model [55]. The theory proposes that environmental factors are the dominant cause of obesity, and that epigenetic intrauterine programming plays the critical role [58,59]. This theory has been refined in the predictive adaptive response model. In this supplement to the original theory, the relative difference in nutrition between prenatal and postnatal environment, rather than an absolute level of nutrition, determines the risk of IR [60]. Both thrifty phenotype theories are accepted in IR research because they have been confirmed in many studies in humans and animals. These days, it is amazing that a nongenetic theory has received so much support and attention.

Based on the thrifty genotype hypothesis, IR and immune activation were recognized as an adaptive positively selected program to combat infections (the fight infections theory of IR). The activation of the immune system during infectious disease and inflammation induces IR, which leads to redirection of glucose to the activated immune system [61]. In a modern form, this was integrated into the concept of immune cell activation by pathogen-sensing and nutrient-sensing pathways (with cytokines, toll-like receptors, jun-N-terminal kinase, and so forth) [62]. Here, even nutrients can induce an inflammatory state that can support IR, which is probably a dilemma after exaggerated food intake when nutrients cannot be adequately stored in fat tissue and elsewhere (nutrient overflow problem).

Similarly based on the thrifty genotype theory is the breakdown of robustness theory, which states that a robust glucose control system developed during evolution. The breakdown of this robust glucose control system induces positive disease-stabilizing feedback loops leading to IR. The critical determinant of the breakdown is TNF [63]. This theory incorporates many accepted aspects but TNF is not the sole pathophysiological factor.

With the discovery of leptin, a negative feedback loop between adipose tissue and food intake was discovered. While in earlier times many argued that energy homeostasis operates primarily to defend against weight loss, the discovery of the leptin negative feedback loop speaks for homeostatic mechanisms that inhibit uncontrolled weight gain. The central resistance model states that central hypothalamic pathways are defective (resistant to leptin and others such as insulin). This leads to increased food intake and the resulting obesity induces IR [64]. This theory has much value because it added the central regulation of food intake to the peripheral pathophysiologic pathways.

Finally, the good calories-bad calories theory explains that our present food is markedly different from paleolithic food. Particularly, high energy-dense carbohydrates are consumed too often, which induces inadequate hyperinsulinemia [65]. Long-term hyperinsulinemia is the platform for obesity and disease sequelae. Others hypothesized that disparities between paleolithic and contemporary food might be important factors underlying the etiology of common western diseases [66]. Typically the type of ingested lipids and the relative amount of carbohydrates/lipids versus proteins is a problem.

In conclusion, the theories already indicate that IR can be an important aspect to support the brain and the activated immune system. As such, IR can be seen as a positively selected program to support the brain or immune system. In the following sections, this concept is further developed by including aspects of energy regulation.

### Energetic benefits of insulin resistance for non-insulin-dependent tissue

At this point, I recapitulate that IR increases circulating glucose and free fatty acids that are not taken up in adipose tissue, liver, and muscle, and are now freely available to all non-insulin-dependent tissues. The two main profiteers of hyperglycemia are the central nervous system and the immune system because either glucose, free fatty acids (not the brain), or ketone bodies are energetic substrates. Both of these organs do not become insulin resistant. In contrast, the immune system profits from insulin because it is an important growth factor for leukocytes and, with the help of insulin, major glucose transporters like glucose transporter-3 and glucose transporter-4 are upregulated on all leukocyte subpopulations [67]. In answering the question of whether, for example, hepatic glucose production really provides higher levels of circulating energy, the following simple calculations are presented for glucose (similar calculations can be done for free fatty acids).

One important factor of IR is overproduction of hepatic glucose [68]. In normal subjects, hepatic glucose production after an overnight fast is approximately 2.0 mg/kg per minute. Under a situation involving IR, for example in T2D patients, insulin is 2.5-fold increased and the rate of fasting glucose production can increase to 2.5 mg/kg/minute [68]. After an overnight fast during an observation period of 12 hours, the liver of a normal person of 80 kg bodyweight produces 115 g glucose. Using the above given numbers, a person with IR produces 144 g glucose, leading to an increase of 29 g in 12 hours. An increase of 2 × 29 g = 58 g glucose in 24 hours corresponds to 974 kJ in 24 hours, which is a pretty high number in the light of the normal metabolic rate of 10,000 kJ/day of an 80 kg person (sedentary way of life). Indeed, 974 kJ represents approximately 39% of the total energy need of the normally active central nervous system, or it represents 61% of the energy requirements of all resting immune cells (Table 5). IR is thus a perfect way to support the activity of the central nervous system, the immune system, and/or other insulin-independent tissues (for example, the heart; Table 5).

**Table 5:** Energy expenditure of systems and organs under sedentary conditions (approximately 10,000 kJ/day)^a ^[69,70,136-139].

System/organ	Energy expenditure per day (kJ/day)
Muscle at rest^b^	2,500
Central nervous system (brain and spinal cord)	2,500
Immune system in a quiescent state^c^	1.600
Liver^d ^(including immune cell activity)	1,600
Heart^b^	1,200
Gastrointestinal tract (including gut immune system, without liver, kidney, spleen)^d^	620
Kidneys	600
Spleen (erythrocytes plus leukocytes; 90% anaerobic)	480
Lungs^d ^(including lung immune system)	400
Skin^d ^(including skin immune system)	100

^a^10,000 kJ = 2,388 kcal. ^b^Activated muscle has a much higher metabolic rate: for example, a Tour de France bicyclist needs approximately 30,000 kJ/day, which is 20,000 kJ more than under sedentary conditions. The 20,000 kJ are used predominantly by the muscles and also the heart. At the upper limit of gastrointestinal resorption, the total body daily uptake (absorptive capacity in the gut) is 20,000 kJ/day. ^c^Moderate activation of the immune system increases daily energy needs to approximately 2,100 kJ/day, and strong activation increases the daily need to 3,000 kJ/day. ^d^Energy need is difficult to estimate independent of the immune system in some organs.

In conclusion, while IR is most often regarded as a pathological state to be treated, these numbers and the fact that IR is linked to so many diseases and disease states are indicative of a beneficial role of IR. While the value of IR can be estimated from the abovementioned numbers, the generation of the two disease clusters is not yet clear.

### The selfish brain and the selfish immune system independently demand energy

This section demonstrates aspects of hypothetical character, and the reader is advised to critically judge the theoretical model. The basal metabolic rate of the entire body is determined when the following conditions are met [69]: awake, lying, after overnight fast, thermoneutral (no heat production due to low/high temperature), and no emotional stress [69]. Under these conditions, a person weighing 80 kg and 1.80 m in height needs approximately 10,000 kJ/day (Table 5).

The so-called minimal metabolic rate is lower than the basal metabolic rate because 15% of energy is spared during sleep, so that a 24-hour sleeping person weighing 80 kg and 1.80 m in height needs 8,500 kJ/day. This amount of energy is not up for negotiation between the different organs. The delta value between this last number and the maximum of daily energy uptake in the gut (20,000 kJ/day; see Table 5) is 11,500 kJ/day. In this example, 11,500 kJ/day is the controllable amount of energy (CAEN) because allocation of the CAEN to different organs is controlled by the interplay of these organs. This amount of energy is available for negotiation. The question is which organs are dominant in regulating the CAEN. Dominance can be judged when looking at Table 5, which shows the main users of energy, but can also be derived from simple theoretical considerations.

For example, if a paleolithic hunter experiences tissue trauma with infection, the immune/repair system becomes strongly activated. In this life-threatening situation, regulation of CAEN allocation to the immune/ repair system must be independent of other organs and immediate (hierarchically, the highest level of control to survive). In this situation, circulating cytokines and activated sensory nerve fibers are responsible for the immediate reallocation of the CAEN to the activated immune system that increases energy consumption (Table 5) [70]. This reaction is called the energy appeal reaction [70].

Similarly, if the brain is active during hard forest work over 6 hours, for example, then the skeletal muscles, heart, lungs/diaphragm, and liver are also active, but most other organs are at minimal metabolic levels. This is particularly true for the gastrointestinal tract and the immune system. In this example of 6-hour forest work, a person weighing 80 kg and 1.80 m in height would need 18,500 kJ for the entire day (calculated using data from [71]). The brain controls the additional CAEN of 10,000 kJ when there is need for forest work. Likewise, if a paleolithic hunter needs to escape from a severe dangerous threat, the brain must control the CAEN. In such a life-threatening situation, the control of the CAEN by the brain must be independent of other organs (again, the highest level of control to survive).

With trauma/infection or fight/flight response, the activity of most organs depends on either the immune/ repair system or the central nervous system, respectively. We recently delineated that allocation of CAEN to the brain and muscles happens mainly during daytime, while allocation of CAEN to the immune/repair systems happens at night [70]. This circadian allocation of energy-rich substrates is another clear indication of tight energy regulation. From these theoretical considerations, it becomes clear that either the immune/repair system or the central nervous system is a dominant regulator of the CAEN.

Coming back to the Introduction, with this model the two clusters of clinical entities linked to IR become understandable in terms of energy regulation. One recognizes two independent organs - the selfish immune system, and the selfish brain [37,72] - related to the abovementioned clusters of inflammation with an activated immune/repair system and of increased mental activation.

With the chronic inflammatory and chronic mental diseases that induce IR (listed in Table 1), the question arises of whether or not brain-supporting and immune system-supporting IR has been positively selected for acute disease or chronic disease. Such a distinction is not included in the available theories of IR, but it might be helpful to understand the role of IR in general.

## A difference between acute and chronic disease

While an acute response is often adaptive and physiological to correct alterations of homeostasis, a chronic disease process is often accompanied by the wrong program [70,73]. Looking at simple readout parameters, this can be demonstrated for immune/repair system activation and mental activation.

The acute activation of the immune/repair system is outstandingly important to fight acute infections and trauma. However, longstanding inflammation in CIDs leads to severe disease sequelae as summarized recently [70,73]. The following disease sequelae are directly linked to CIDs: sickness behavior, anorexia, malnutrition, muscle wasting-cachexia, cachectic obesity, IR with hyperinsulinemia, dyslipidemia, increase of adipose tissue near inflamed tissue, alterations of steroid hormone axes, elevated sympathetic tone and local sympathetic nerve fiber loss, decreased parasympathetic tone, hypertension, inflammation-related anemia, and osteopenia [70,73]. It was suggested that these sequelae of CIDs are a consequence of a high energy demand of the activated immune/repair system accompanied by water retention [70,73]. Acute activation of the immune/ repair system can be very helpful, but chronic activation is a harmful process that worsens the situation in an affected patient.

Considering mental activation, we can also separate acute versus chronic. In the acute situation of emergency for a loved one, family members and hospital staff show strong mental activation that can lead to a higher state of activity, a better readiness to take action, but also poor sleep and symptoms of anxiety [74,75]. Similarly, student's examination stress can lead to a higher state of activity but also to poor sleep and acute increase in anxiety scores [76,77]. Acute examination stress increased intake of highly palatable food in an unproportional manner [78]. In these acute situations, mental activation, poor sleep, and increase in food intake are important to overcome the challenging situation.

However, long-term caregivers of, for example, Alzheimer disease patients are more often obese than noncaregivers, demonstrate alterations typical of the metabolic syndrome, show a higher risk to develop major depression, and have a long-term increase in proinflammatory markers [79-84]. Similarly, chronically stressed students in a highly competitive university environment showed an increased risk of obesity [14]. A dose-response relationship was found between chronic work stress and risk of general and central obesity that was largely independent of covariates such as age, sex, and social position [11], supported in other large studies [12,13]. Moreover, chronic job stress was related to an increased risk of the metabolic syndrome and even T2D [85-87]. Chronically poor sleep is related to metabolic risk factors, obesity, and inflammation [88].

This small collection demonstrates that activation of the immune/repair and central nervous systems are successful in acute emergency, but dangerous when applied chronically, leading to typical signs of obesity, metabolic derangement with IR, chronic inflammation, and increased risk for cardiovascular events [89]. The question is why there is such a clear distinction between acute and chronic, which determines the full picture of the metabolic syndrome and IR.

## Evolutionary medicine - acute physiological response versus chronic disease

Earlier, it was demonstrated that a highly activated immune/repair system cannot be switched on for a long time because this would be very energy consuming [73]. A highly activated immune system is accompanied by sickness behavior and anorexia, which prevents adequate food intake and necessitates life on stored reserves (inflammation-induced anorexia). Under systemic inflammatory conditions, breaking down all reserves takes 19 to 43 days [73]. A highly activated immune/repair system can need huge amounts of energy, which is exemplified in the case of extensive burn wounds (up to 20,000 kJ/day) [73]. Although this aspect demonstrates the extreme of the spectrum, it indicates that energy consumption is a critical factor during evolution.

I hypothesize that energy consumption and energy protection are the most critical determinants in evolution, to undergo either negative selection or positive selection, respectively. If alterations of homeostasis lead to marked energy consumption, the situation cannot be chronic - it must be acute. Since the total consumption time ranges between 19 and 43 days [73], an acute energy-consuming change of homeostasis must be started and terminated in this time frame. A very good example for this time window is the germinal center reaction of B-lymphocyte expansion and contraction that happens within approximately 21 to 28 days [90]. Most acute disease states are terminated within this time frame, such as infectious diseases, wound healing, and repair, but also strong mental activation in stressful situations must be terminated because they are energy consuming, exemplified in short-term stress [78]. During evolution, respective homeostatic networks were positively selected for short-lived, acute, energy-consuming responses but not for longstanding polygenic CIDs or chronic mental illness. These chronic situations generated a huge negative selection pressure.

In contrast, if mutations were helpful to protect energy reserves, they were positively selected during evolution. This is true for memory responses because immediate reaction of an educated system can spare energy reserves. This is exemplified by the immune memory that leads to shorter, more effective and, finally, less energy-consuming reactions towards microbes. Importantly, acquisition of immune memory during the primary contact must fit into the above specified time frame of 19 to 43 days (and this happens as exemplified by the germinal center reaction in secondary lymphoid organs). In this context, tolerance versus harmless foreign antigens of microbes on body surfaces (see gut, skin, respiratory tract, urogenital tract) or harmless autoantigens is a memory function that spares energy reserves. Sometimes microbes such as *Mycobacterium tuberculosis, Mycobacterium leprae*, and viruses enable or mimic tolerant immune responses leading to longstanding infection, but finally leading to death due to emaciation.

Similarly, neuronal memory can largely decrease time to accomplish successful foraging in the wild [91]. Neuronal memory systems are tuned to ancestral priorities in the context of foraging and other paleolithic tasks [92]. Additionally, tool-making, invention of language and writing, and storage of data on computer hard disks protects time and thus energy.

Another example of positively selected gene variants is observed for food ingestion and fat storage (not IR!), both of which are important in determining the above-mentioned consumption time. Indeed, female *Australopithecus afarensis *had a consumption time of approximately 19 days, while modern female *Homo sapiens *can rely on 43 days [73]. Particularly, fat storage has markedly increased over the last 3 to 4 million years of human evolution. Not surprisingly, the latest metaanalysis of genome-wide association studies of obesity and the metabolic syndrome (not IR) found polymorphisms in genes relevant for food intake such as *FTO *(fat mass and obesity related), *MC4R *(melanocortin receptor type 4), *POMC *(proopiomelanocortin, the precursor of melanocortin), and genes relevant for fat storage such as the insulin-stimulating *GIPR *(gastric inhibitory polypeptide receptor) [93].

Another important indication for positive selection of fat storage networks (not IR) is given by the fact that the number of adipocytes in humans is determined before puberty [57]. After puberty, the number of adipocytes stays constant with an annual exchange rate of 10% [57]. If spontaneous mutations lead to a phenomenon relevant before reproduction time, it will be easily transferred to offspring when it is an advantageous trait. Since the phenomenon still exists in modern children [57], we expect that fat storage was an important factor during evolution. Similarly, humans can deposit large amounts of fat *in utero *and are consequently one of the fattest species at birth [94]. In addition, newborn humans devote roughly 70% of growth expenditure to fat deposition during early postnatal months, which reduces the risk of energy stress during infections [94]. If the newborns are not able to store large amounts of fat tissue *in utero*, or if malnutrition is a problem in fetal life (thrifty phenotype model, see above; Table 4), a postnatal program seems to be switched on that supports obesity during childhood and adolescence [55,56]. Again this is an indication that important positively selected gene variants exist that serve storage of energy.

**Figure 1:**
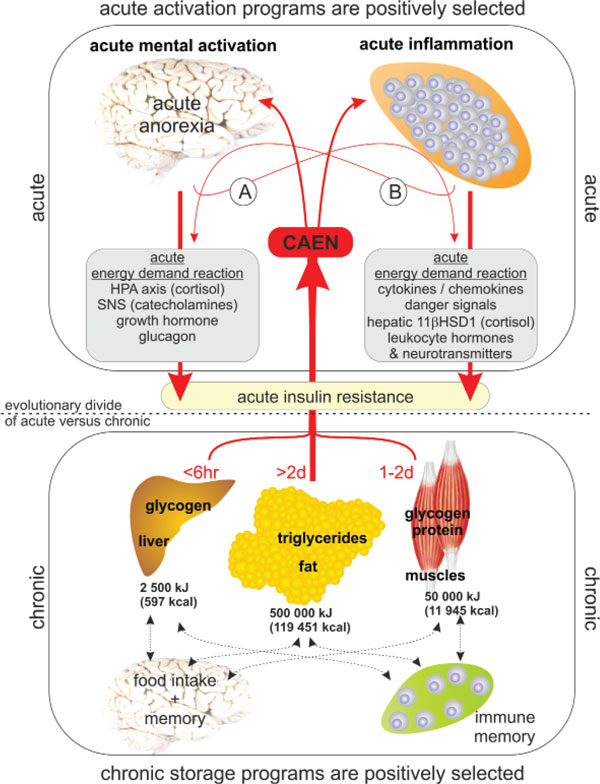
**Pathophysiology of insulin resistance according to the new theory**. Upper panel: Acute activation programs were positively selected for short-lived activation of either the brain or the immune system. Hierarchically, the brain and the immune system are on the same level. Activation of the brain mainly stimulates stress axes hormones and activates the sympathetic nervous system (SNS). This is supported by a mild inflammatory process that is paralleled by mental activation (A). Activation of the immune system induces cytokines, chemokines, and danger signals. In addition, the inflammatory process uncouples the locally inflamed area from the control of the brain by cytokine-induced hormone/ neurotransmitter production in the periphery independent of superordinate stress pathways. This leads to hepatic cortisol secretion [140], adrenocorticotropic hormone-independent cortisol secretion [141], and production of leukocyte hormones [142] and leukocyte neurotransmitters [143]. The activation of the immune system is accompanied by a mild stimulation of the hypothalamic-pituitary-adrenal axis (HPA) axis (albeit inadequately low in relation to inflammation) and a somewhat stronger stimulation of the SNS (B). Despite activation of the SNS, anti-inflammatory neurotransmitters of sympathetic nerve fibers do not reach the uncoupled inflamed tissue [144]. Inflammatory and mental activation are often accompanied by anorexia and sickness behavior, which aggravates energy shortage. Lower panel: Chronic energy storage and memory programs were positively selected. The major storage organs are fat tissue (glycerol, free fatty acids) and muscles (proteins). The liver is more a switchboard to interchange and renew energetic substrates. The main storage factor is insulin so that insulin resistance can be seen as a catabolic program induced by catabolic pathways (upper panel). Numbers in red give the typical time of energy provision by the respective organ (amino acids from muscle are spared from day 3 onwards). Storage is mainly supported by a positively selected program of foot intake/foraging behavior and memory. Memory is outstandingly important to spare energy-rich fuels (brain, immune system). Dashed black arrows in the lower panel demonstrate real and hypothetical connections between respective organs. Black numbers give a typical figure of stored energy in the respective organs. Dashed black line between upper and lower boxes separates the programs positively selected for acute (catabolic) versus chronic states (storage and memory). CAEN, controllable amount of energy (the energy that is regulated and negotiated between organs); 11βHSD1, 11-beta-hydroxy steroid dehydrogenase type 1 [140].

In conclusion, networks are positively selected if they serve acute, highly energy-consuming situations, which are terminated within 3 to 6 weeks. We perceive a chronic disease when it lasts for longer than 6 weeks, as used in classification criteria in RA and juvenile idiopathic arthritis [95]. In addition, gene variants are positively selected if they protect energy stores, which is relevant during the entire life (beyond weeks 3 to 6). Networks that lead to IR serve the acute activation of the selfish immune system or the selfish brain, but do not belong to networks that protect energy stores (Figure 1). In contrast, IR leads to loss of energy-rich substrates because it is a catabolic process (energy-rich fuels are consumed by non-insulin-dependent organs or are simply excreted) (Figure 1). If the hypothesis of the acute IR program is correct, then chronic IR in chronic inflammation, in CIDs, and in chronic mental activation or mental disease is a misguided acute program. In contrast to IR, food intake and storage of energy-rich substrates in adipose tissue *per se *is not a misguided program. In other words, obesity is not dangerous and obesity is not a disease [96]. Yet obesity becomes a problem if additional factors are switched on that usually serve acute energy-consuming situations (mental activation or inflammation). Per Björntorp once noticed that 'some disease-generating factors, in addition to the basic condition of central obesity, is required for associated diseases to become manifest' [96].

## The new model of insulin resistance

With all this information, one can generate a new model of IR that builds upon the existing theories. The new model includes four new aspects: it respects much more the immune/repair system, whose energy requirements are enormous (Table 5) [70]; it juxtaposes the selfish brain and the selfish immune system on a similar hierarchical level in terms of energy demand and requirements (Table 5); it respects that energy requirements convey an evolutionary pressure (highly energy-consuming states are acute (negative selection pressure), energy storage is beneficial (positive selection pressure)); and it accepts that either immune system activation or mental activation are equally important in inducing IR. On the basis of these elements, a new model of IR is presented in Figure 1. This model states that IR is an acute catabolic program to serve the selfish immune system or the selfish brain, positively selected for inflammation with an activated immune/repair system and for increased mental activation.

Several testable hypotheses can be generated from the new model, as follows. Obesity is only a problem if acute energy-consuming programs are switched on (either inflammation or mental activation cause the problem). Immunological tolerance should support the storage function of fat tissue and muscle. Nutrient-induced inflammation is only a problem if energy-rich fuels are not properly stored (there is an individual storage threshold). Intrauterine constraints (elements of the thrifty phenotype model) should set the thresholds for acute activation programs. While there is a clear link between fat tissue and brain (leptin), there should be similar pathways between the liver/brain and the muscle/ brain that regulate food intake - concerning the muscle/ brain pair, a recent paper found important links through muscle-derived IL-6 [97]. In CIDs, the selfishness of the immune system should lead to an inhibition of brain-dependent regulation of energy allocation. Likewise, in mental illness or chronic psychological stress, the selfishness of the brain should lead to inhibition of the immune system-dependent regulation of energy allocation. In CIDs and mental illness/stress, the two systems must inhibit each other.

## The drivers of insulin resistance in chronic inflammatory and mental diseases

A seminal study demonstrated the interrelation between the dose of subcutaneously injected recombinant human IL-6, serum levels of IL-6, and the increase of energy expenditure in healthy volunteers [98]. Injection of 0.1 μg recombinant human IL-6/kg bodyweight increased serum levels of IL-6 to approximately 10 to 15 pg/ml, 1.0 μg led to 45 pg/ml, 3.0 μg stimulated a serum level of 250 pg/ml, and 10 μg recombinant human IL-6/kg body-weight was accompanied by an IL-6 serum concentration of more than 1,000 pg/ml. In parallel, the maximal increase of metabolic rate in percent of basal metabolic rate was 4%, 7.5%, 18%, and 25%, respectively [98]. This means that a visible influence on energy regulation was observed at a serum level of 10 to 15 pg/ml, but the effect was small in these healthy volunteers. In contrast, serum levels of 45 pg/ml were related to an increase in metabolic rate of 7.5%, which would amount to approximately 750 kJ/day in a normal-sized healthy subject (basal metabolic rate: 10,000 kJ/day). An increase of serum IL-6 from 1 to 2 pg/ml, as in healthy subjects [99], to 45 pg/ml thus induces a marked energy expenditure program.

Under consideration of the new model in Figure 1, we immediately recognize the problem of continuous inflammation in CIDs. CIDs such as RA are accompanied by markedly elevated serum levels of IL-6 ranging from 40.0 pg/ml before anti-TNF therapy to 8.0 pg/ml after anti-TNF therapy [100]. The levels are thus much higher as compared with healthy subjects (1 to 2 pg/ml [99]). Untreated patients with RA should increase daily energy expenditure by 750 kJ/day (basal metabolic rate: 10,000 kJ/day). This value of 750 kJ/day is remarkably similar to the number of 974 kJ/day obtained by hepatic IR as calculated above. Since we expect that several cytokines like TNF, IL-6, interferon gamma, interferon alpha, and others can drive a similar energy reallocation program, elevation of systemic cytokines explains why patients with CIDs do not need any other factor to provoke IR. These CID patients do not need the activation of the brain and thus activation of stress axes to induce IR. The brain is silenced in CIDs (sickness behavior). IR can be stimulated by a direct influence of cytokines on hepatocytes, adipocytes, and myocytes. We now understand why cytokine-neutralizing therapies work perfectly well in RA - because the key IR factor is removed. When cytokine-neutralizing strategies do not work in obese or T2D people, other parallel factors must play an enormous role.

The inflammatory load is remarkably different in the situation of chronic mental illness or psychological stress where mild peripheral inflammation probably plays a small supportive role. When one compares serum levels of IL-6 as measured with the identical quantitative high-sensitivity enzyme-linked immunosorbent assay technique, healthy subjects range between 1 and 2 pg/ml [99], caregivers show a mean value of 5.5 pg/ml [101], and subjects who report a high level of perceived hopelessness show 3.0 pg/ml [102]. These levels correspond to mild activation of the immune system, but they would not lead to an energy reallocation program [98]. Thus, in mental activation, stress axes must play the major role for the observed IR (cortisol, adrenaline, growth hormone, glucagon). It is expected that neutralization of one cytokine would not change IR in these mentally activated people. Furthermore, when cytokine neutralizing strategies do not work in T2D patients, several factors in parallel are expected to drive IR. It is interesting that salsalate had a positive impact on IR in T2D [43], but this type of drug and other nonsteroidal anti-inflammatory drugs can also inhibit mental activation in various chronic psychiatric diseases [103-105], which is most probably related to reduced activation of stress axes.

## Conclusions

IR is an unfavorable factor in CIDs because it supports the already activated immune system. IR is a direct consequence of the proinflammatory load. Thus, IR should be treated by neutralizing inflammatory cytokines or by inhibiting the immune system with disease-modifying anti-rheumatic drugs in a more general way (like salsalate for T2D). Since IR is a very direct consequence of immune system activation, the primary goal is anti-inflammatory treatment. In CIDs, further treatment of IR beyond good inflammatory control is expected not to be needed. Since IR is a perfect diagnostic marker of an activated energy reallocation program (inflammation and mental activation), measuring IR might be a suitable biomarker to study the control of systemic inflammation in CIDs. Since several cytokines induce IR in a redundant manner, IR might be a more integral systemic diagnostic marker than C-reactive protein, the erythrocyte sedimentation rate, or single cytokines.

In addition to aspects of IR in CIDs, this review demonstrates an extended theory of IR that classifies IR as a beneficial positively selected program to support activation of the immune/repair system and the brain. IR makes sense in acute alterations of homeostasis in the context of short-lived diseases but is a misguided program in long-term inflammatory and mental activation.

## Key messages

• IR is a consequence of mental activation (neuroendocrine axes) or inflammation that is a consequence of selfishness of the brain or the immune system.

• IR has been positively selected during evolution for short-lived energy-consuming activation of the brain or immune system.

• Long-term IR supports mental disease and CIDs because energy-rich fuels are provided to these non-insulin-dependent tissues (continuous activation).

• IR in CIDs is treated by consequent reduction of the proinflammatory load.

• Treatment of IR in morbid obesity and T2D is more complex because both inflammatory and neuroendocrine pathways need to be targeted. The pleiotropic anti-inflammatory and central nervous effects of salsalate constitute the first positive drug therapy of IR in T2D.

## Abbreviations

CAEN: controllable amount of energy; CID: chronic inflammatory disease; IL: interleukin; IR: insulin resistance; RA: rheumatoid arthritis; T2D: type 2 diabetes mellitus; TNF: tumor necrosis factor.

## Competing interests

The author declares that they have no competing interests.

## Declaration

This article has been published as part of *Arthritis Research & Therapy* Volume 16 Suppl 2, 2014: At the interface between immunology and endocrinology in rheumatic diseases. The full contents of the supplement are available at http://arthritis-research.com/supplements/16/S2.

This supplement was proposed, developed and commissioned by *Arthritis Research & Therapy *and was funded by an educational grant from Horizon Pharma Inc. All published articles were independently prepared by the authors and have undergone peer review in accordance with the journal's standard policies and processes. Horizon Pharma Inc had no input into the topics covered or the articles themselves. The Supplement Editor was appointed by the journal and declares that they have no competing interests.
